# Management of first‐time shoulder dislocations: A survey of sport medicine physician perceptions

**DOI:** 10.1002/ksa.70297

**Published:** 2026-02-06

**Authors:** Danielle Dagher, Ethan Mewhinney, Peter MacDonald, Rachel M. Frank, Xinning Li, Wade Elliott, Katie Dalziel, Moin Khan

**Affiliations:** ^1^ Department of Health Research Methods, Evidence, and Impact McMaster University Hamilton Ontario Canada; ^2^ Faculty of Health Sciences McMaster University Hamilton Ontario Canada; ^3^ Department of Surgery, Section of Orthopedics University of Manitoba Winnipeg Manitoba Canada; ^4^ Department of Orthopaedic Surgery University of Colorado School of Medicine Aurora Colorado USA; ^5^ Department of Sports Medicine and Shoulder Surgery Boston University School of Medicine Boston Massachusetts USA; ^6^ Department of Family Medicine McMaster University Hamilton Ontario Canada; ^7^ Division of Orthopaedic Surgery, Department of Surgery McMaster University Hamilton Ontario Canada

**Keywords:** dislocation, instability, shoulder, sport physicians, survey

## Abstract

**Purpose:**

The purpose of this study was to survey sport medicine physicians globally to evaluate how they treat patients with a first‐time shoulder dislocation (FTSD), specifically exploring the most common management strategies, the evidence or guidelines guiding these decisions, and the influence of demographic factors on these strategies and perceptions.

**Methods:**

A cross‐sectional survey was developed and distributed globally from 14 October 2024 through 22 March 2025 to sport medicine physicians involved in the management of shoulder instability. The questionnaire assessed respondents' demographics, preferred management strategies for FTSDs, and perceptions regarding the evidence supporting various treatment approaches. Descriptive statistics were used to summarize the data, and regression analyses were conducted to explore associations between demographic variables and management practices and perceptions.

**Results:**

A total of 326 respondents completed the survey, predominantly fellowship‐trained orthopaedic surgeons from North America and Europe. The most common management strategy was immobilization for 1–3 weeks, followed by physical therapy incorporating strengthening and proprioception exercises. Imaging practices varied geographically, with North American respondents preferring radiographic assessment and Europeans favouring magnetic resonance imaging at initial presentation. Surgical management or consideration for surgical intervention was most influenced by the presence of bony injury (81%), patient age (69%) and participation in contact sports (63%). Younger physicians favoured arthroscopic stabilization, while older respondents leaned towards open approaches.

**Conclusion:**

While variability in the management of FTSDs persists, the results of this study demonstrate emerging trends towards consensus on critical factors for consideration in the management of such patients. However, despite the prevalence of guideline‐based management, a substantial proportion of respondents, particularly those over 55 years old, continue to base their practices on personal experience and training rather than standardized protocols. The development and dissemination of evidence‐based guidelines remain essential to standardize clinical practices and optimize patient outcomes.

**Level of Evidence:**

Level IV.

**Abbreviations**
FTSD: first‐time shoulder dislocation
GBL: glenoid bone loss

## INTRODUCTION

The shoulder is the most commonly dislocated joint in the body, with a global incidence ranging from 15 to 25 per 100,000 people [7, 11, 14]. It is estimated that the annual societal cost in North America due to first‐time shoulder dislocations (FTSDs) exceeds $1.2 billion Canadian dollars (CAD) annually, or over $14,000 per capita [7, 8]. The cost per shoulder dislocation is high, particularly given the high risk of recurrent dislocations and the timing of injury in youthful years. Investment in research to decrease cost and improve the quality of care is of high priority.

Following the initial urgent management and relocation of a dislocated shoulder, the prevention of recurrent instability is the critical management consideration for healthcare providers. Two initial management options exist: non‐operative or operative intervention. The current standard of care for the vast majority of FTSDs is a brief period of immobilization followed by physiotherapy [10]. Failure of non‐operative management with a recurrent dislocation or further instability remains the indication for surgical stabilization [5]. Proponents of the non‐operative approach argue that approximately 50% of patients are adequately treated by non‐operative methods alone without additional risks of surgery [2, 6]. Proponents of the primary surgical stabilization approach argue that evidence for early non‐operative treatment is inconclusive, recurrent instability rates are unacceptably high and delay in treatment results in further injury to the joint [4, 5]. Substantial variability exists not only between but also within non‐operative and operative practices, including the type and duration of immobilization as well as the optimal surgical treatment. There are advocates for both treatment options, and no consensus on which produces better outcomes for these patients due to a lack of evidence. This lack of agreement highlights the need to better understand how sports medicine physicians approach FTSDs in practice.

The purpose of this study was to survey sport medicine physicians globally to evaluate how they treat patients with an FTSD, specifically exploring the most common management strategies, the evidence or guidelines guiding these decisions and the influence of demographic factors on these strategies and perceptions. We hypothesize that variability exists in the management of FTSDs among sport medicine physicians, influenced by certain physician characteristics and practice settings.

## METHODS

### Survey development

This cross‐sectional survey was developed by a focus group consisting of four fellowship‐trained orthopaedic surgeons (P.M., R.M.F., X.L. and M.K.), two sports medicine physicians (W.E. and K.D.) and a research methodologist (D.D.). Questions were developed to examine respondents' demographics (age, years in independent practice, primary specialty, fellowship training, practice location, etc.), management preferences for treating FTSDs (duration of immobilization, when to initiate physical therapy, type of physical therapy recommended, imaging modalities used, etc.) and perceptions regarding existing evidence and guidelines (guidelines used to guide practice, opinion on quality of available evidence, etc.) The survey was then pre‐tested prior to distribution to ensure face and content validity. The final questionnaire consisted of 21 multiple‐choice questions. A complete copy of the questionnaire is available in Appendix SA.

### Ethical considerations

The study protocol was approved by the Hamilton Integrated Research Ethics Board (HiREB; project ID 17955) prior to distribution of the survey. Consent was obtained in the form of a statement indicating that by completing the survey, the participant consents to the use of their data for analysis. Participants had the option to exit the survey at any point prior to submission. All responses were anonymous.

### Survey administration

Ninety‐nine orthopaedic sports medicine organizations were individually contacted by email, inviting all members to participate in the survey. The survey was administered electronically via the REDCap (Research Electronic Data Capture) system from 14 October 2024 through 22 March 2025. Restrictions were in place to ensure only one response per computer. Only sports medicine physicians managing shoulder instability were eligible to complete the survey. All involved organizations agreed to distribute the survey at least twice to their membership to maximize the response rate following the initial email.

### Data analysis

Descriptive statistics (frequency and percentage distributions) were used to summarize the data. The chi‐square test of independence was used to assess the relationships between independent (demographic) and dependent (outcome) variables. For binary outcome variables, binomial logistic regression was used to evaluate the influence of multiple demographic variables. For outcome variables with more than two categories, multinomial logistic regression was used to model the relationships with multiple predictors. As this was an exploratory survey, no formal correction for multiple comparisons was applied.

## RESULTS

Of the 99 organizations that were contacted to distribute the survey, 21 replied. Thirteen organizations agreed to participate in the survey, while six said they were unable due to their organization's survey distribution guidelines and two organizations deferred the request to other organizations. A complete list of organizations contacted, and their response status, is available in Appendix SB. Overall, a total of 326 respondents completed the survey, representing approximately a 2% response rate.

### Respondent demographics

**Figure 1:**
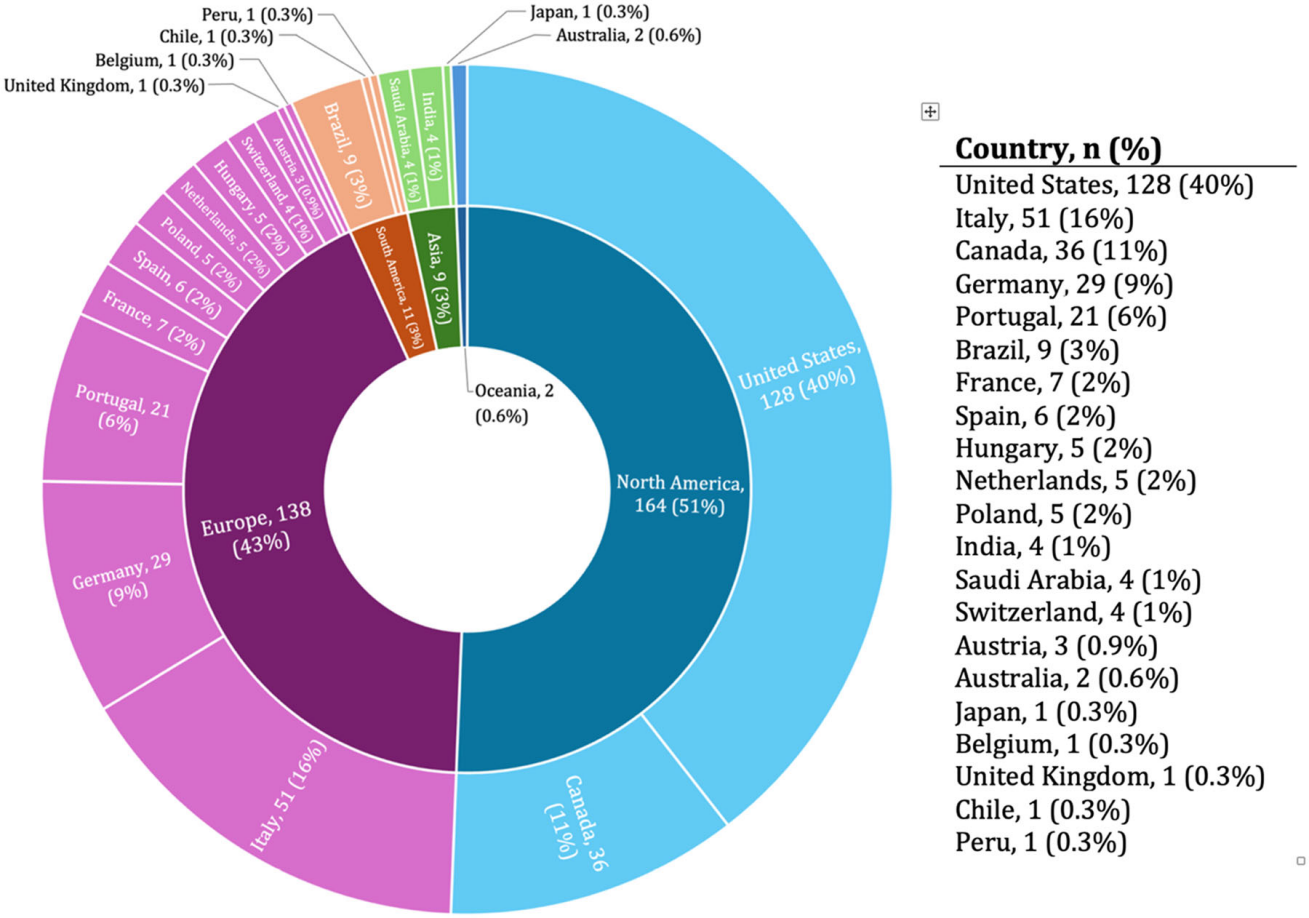
Respondents' geographical location.

The majority of survey respondents (62%) were between the ages of 36 and 55, 31% were older than 55, 7% were 25–35, and none were under 25 (Table 1). Most (65%) had been in independent practice for more than 10 years, including 25% practicing for over 25 years. Only 13% of respondents had been in practice for less than 5 years (Table 1). Orthopaedic surgery was the primary speciality for nearly all respondents (95%), with very limited representation from other fields (Table 1). Fellowship training was common among orthopaedic surgeons, with 84% having completed post‐graduate subspecialty training; 63% in shoulder and elbow surgery and 36% in sports medicine (Table 1). Respondents practiced in various countries (*n* = 21), but most were from the United States (39.5%), Italy (15.7%), Canada (11.1%), Germany (9%) and Portugal (6.5%) (Figure 1).

**Table 1:** Respondent demographics and practice characteristics.

Question	*n* (%)
Age (years), *n* = 326	
18–24	0 (0)
25–35	22 (7)
36–45	111 (34)
46–55	92 (28)
>55	101 (31)
Years in independent practice, *n* = 326	
<5	43 (13)
5–10	72 (22)
11–20	85 (26)
21–25	45 (14)
>25	81 (25)
Primary specialty, *n* = 326	
Orthopaedic surgery	310 (95)
Primary care sports medicine	13 (4)
Physical medicine and rehabilitation	1 (0.3)
Emergency medicine	1 (0.3)
Physician assistant/nurse practitioner	0 (0)
Other	1 (0.3)
Formal post‐graduate fellowship training among orthopaedic surgeons, *n* = 310	
Yes	259 (84)
Sports medicine fellowship	94 (36)
Shoulder and elbow fellowship	164 (63)
No response	1 (0.4)
No	50 (16)
No response	1 (0.3)
Patients commonly seen in practice, *n* = 326	
Recreational athletes	258 (79)
General outpatient orthopaedic patients	231 (71)
High school athletes	137 (42)
Seniors	125 (38)
College athletes	124 (38)
Professional/elite athletes	72 (22)
Paediatrics	17 (5)
Other	8 (2)
FTSDs managed in practice annually, *n* = 325	
0	2 (0.6)
1–5	35 (11)
6–10	78 (24)
11–20	97 (30)
>20	113 (35)
Time to first consultation following an FTSD, *n* = 323	
<1 week	74 (23)
1–2 weeks	117 (36)
2–6 weeks	107 (33)
6–12 weeks	18 (6)
>12 weeks	7 (2)

Abbreviation: FTSD, first‐time shoulder dislocation.

### Respondents' practice characteristics

Regarding patient populations, the majority worked with recreational athletes (79%) and general outpatient orthopaedic patients (71%). A smaller portion reported working with high school athletes (42%), collegiate athletes (38%) and professional/elite athletes (22%) (Table 1). Nearly all respondents (99%) reported treating patients with shoulder dislocations. A majority (65%) managed more than 10 FTSD cases per year, with 35% managing over 20 annually (Table 1). Most respondents (59%) reported seeing these patients within 2 weeks after the dislocation and 33% reported seeing them 2–6 weeks after the dislocation. Only 8% of respondents reported seeing these patients more than 6 weeks after their dislocation (Table 1).

### Management strategies

Most respondents (58%) recommended an immobilization period of 1–3 weeks following an FTSD. A smaller portion (12%) extended immobilization to 3–6 weeks, and only 6% advised no immobilization at all (Table 2). The majority (74%) reported initiating physical therapy after a period of immobilization, whereas 16% began therapy immediately after reduction (Table 2). A large proportion (80%) used a combination of strengthening, range of motion and proprioceptive exercises in their rehabilitation protocols (Table 2). The decision on whether or not to limit external rotation during the early phase of rehabilitation was nearly even, with 55% reporting that they do and 45% reporting that they do not (Table 2).

**Table 2:** Respondent approaches for immobilization and rehabilitation following a first‐time shoulder dislocation (FTSD).

Question	*n* (%)
Duration of immobilization, *n* = 323	
No immobilization	18 (6)
<1 week	77 (24)
1–3 weeks	188 (58)
3–6 weeks	40 (12)
>6 weeks	0 (0)
Initiation of physical therapy, *n* = 323	
Immediately after reduction	52 (16)
After a period of immobilization	239 (74)
Only if there are signs of instability after immobilization	8 (2)
Do not typically refer to physical therapy	21 (7)
Other	3 (0.9)
Physical therapy protocol, *n* = 323	
Strengthening exercises	19 (6)
Range of motion exercises	13 (4)
Proprioceptive training	15 (5)
Combination of all of the above	260 (80)
Do not recommend physical therapy	16 (5)
Limitations to external rotation in the initial phase of physical therapy, *n* = 323	
Yes	178 (55)
No	145 (45)

**Figure 2:**
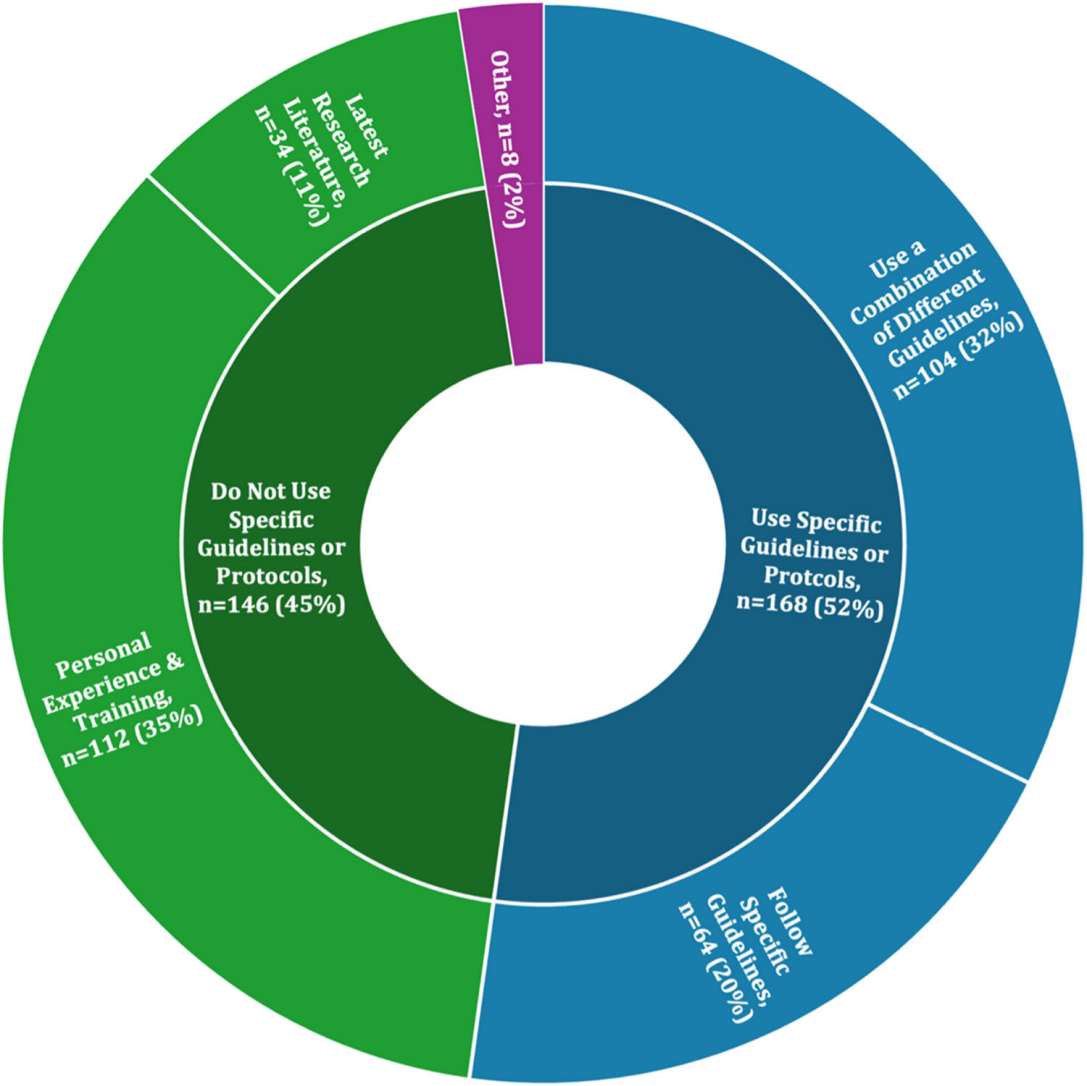
Use of specific guidelines or protocols to guide treatment of first‐time shoulder dislocations (FTSDs).

When asked whether they use specific guidelines or protocols to guide the treatment of FTSDs, 52% of respondents reported that they do, 45% reported that they do not, and 3% selected ‘other’. 20% reported that they follow specific guidelines, 32% reported that they use a combination of different guidelines, 35% reported that they based their approach on personal experience and training, and 10% reported that they rely on the latest research literature. Of the respondents who selected ‘other’, many reported basing their decision on a combination of different patient‐specific factors to individualize care for each patient (Figure 2).

**Figure 3:**
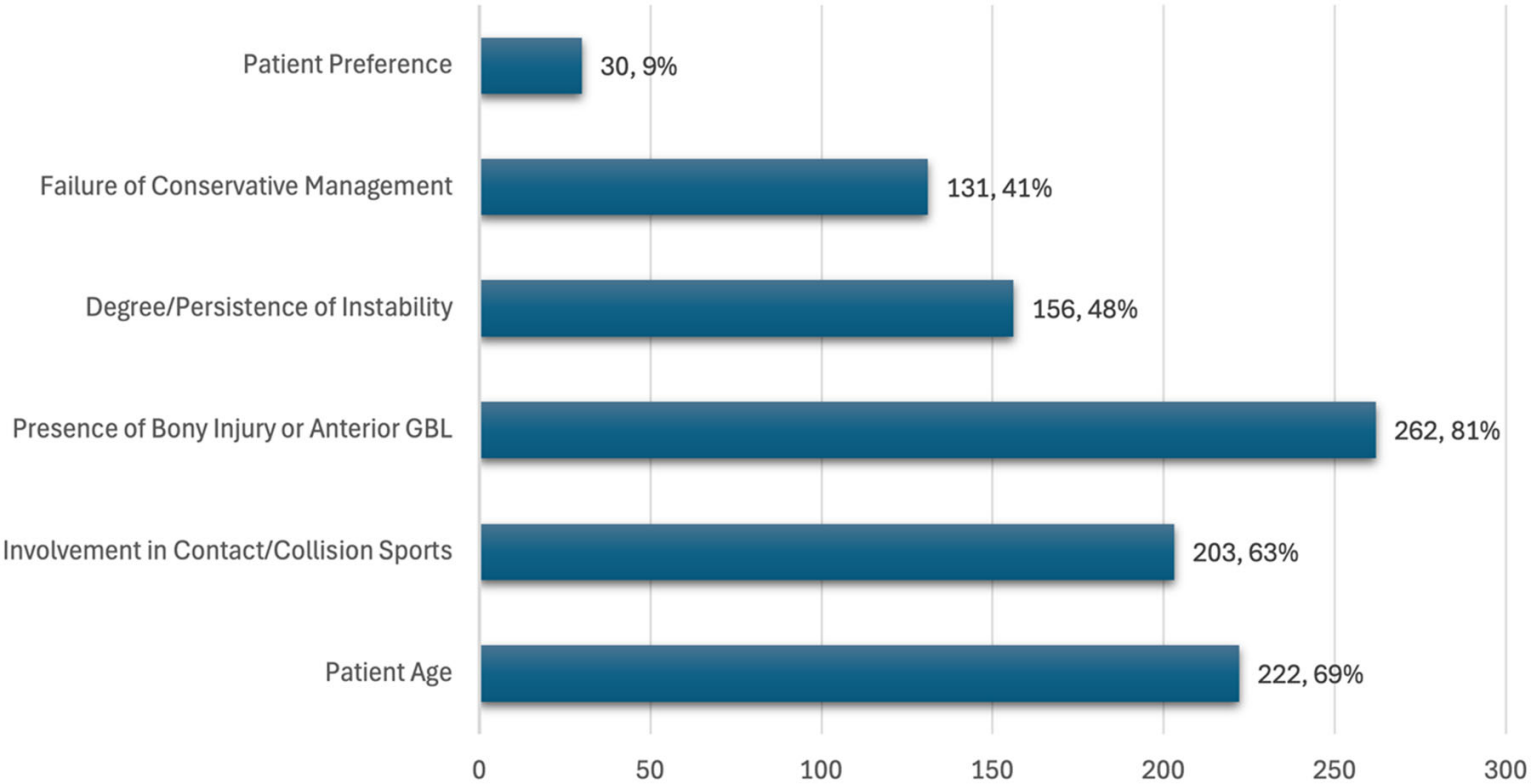
Most important factors influencing surgical management or consideration for surgical intervention for a first‐time shoulder dislocation (FTSD) (*n* = 323). GBL, glenoid bone loss.

Imaging was widely used in the evaluation of FTSDs, with 83% of respondents reporting X‐ray use and 58% reporting magnetic resonance imaging (MRI) use. Computed tomography scans and MR arthrograms were each selected by 18% of respondents, ultrasound by only 2%, and 1% reported not routinely using imaging. When asked about the top three most important factors influencing consideration for surgical intervention for an FTSD, the most important factors selected were the presence of bony injury or anterior glenoid bone loss (81%), patient age (69%), and participation in contact or collision sports (63%). Interestingly, only 9% of respondents reported patient preference as an important consideration (Figure 3).

Of the 326 respondents, 310 were orthopaedic surgeons. Among orthopaedic surgeons, 80% reported performing surgery on FTSDs. Among the orthopaedic surgeons who reported that they perform surgery on FTSDs, in cases of minimal to no bone loss present, 92% selected arthroscopic soft tissue stabilization as their preferred method, 6% preferred open soft tissue stabilization and 2% selected open or arthroscopic bony stabilization (Table 3). Regarding the addition of Remplissage to soft tissue repair, 61% of orthopaedic surgeons reported adding it when an engaging Hill–Sachs lesion was present, and 24% did so for any Hill–Sachs lesion. In the setting of glenoid bone loss (GBL), 21% reported adding Remplissage in cases of GBL less than 10% and 29% reported adding it in cases of GBL greater than 10%. A total of 13% reported that they never add Remplissage, and 7% reported that they add it to every case (Table 3).

**Table 3:** Surgical management of first‐time shoulder dislocations (FTSDs) by orthopaedic surgeons.

Question	*n* (%)
Type of stabilization performed, *n* = 255	
Open soft tissue stabilization	5 (2)
Arthroscopic soft tissue stabilization	235 (92)
Open or arthroscopic bony stabilization	15 (6)
Indication for addition of Remplissage to soft tissue stabilization, *n* = 255	
Hill–Sachs lesion	
Only for engaging Hill–Sachs lesions	156 (61)
Any Hill–Sachs lesion	62 (24)
Glenoid bone loss (GBL)	
GBL < 10%	53 (21)
GBL > 10%	74 (29)
Apprehension/reduced range of motion	10 (4)
Add Remplissage to every case	17 (7)
Never add Remplissage	34 (13)

### Perceptions on evidence and guidelines

When asked about the evidence supporting primary arthroscopic stabilization for FTSDs, most respondents expressed confidence, with 66% either agreeing (40%) or strongly agreeing (26%) that sufficient evidence exists. Meanwhile, 21% neither agreed nor disagreed, 12% disagreed and 2% strongly disagreed (Table 4). To stay current with the latest evidence and guidelines, respondents most frequently relied on peer‐reviewed journals (82%) and professional conferences (81%), followed by continuing medical education (67%), professional networks and consultations (43%) and online medical resources (31%). Only 2% reported using other sources, including discussion with other colleagues.

**Table 4:** Respondent perceptions on evidence/guidelines for treatment of first‐time shoulder dislocations (FTSDs).

Question	*n* (%)
Degree to which respondents agree that sufficient evidence exists to support arthroscopic stabilization for an FTSD, *n* = 326	
Strongly agree	85 (26)
Agree	129 (40)
Neither agree nor disagree	67 (21)
Disagree	39 (12)
Strongly disagree	6 (2)
Adequacy of current guidelines addressing FTSD management, *n* = 326	
They are comprehensive and useful	37 (11)
There is room for improvement	200 (61)
They lack sufficient evidence	43 (13)
They are outdated	27 (8)
Unfamiliar with current guidelines	19 (6)
Consensus or strong evidence for rehabilitation protocols to guide non‐operative management of an FTSD, *n* = 326	
Yes	44 (13)
No	215 (66)
Unsure	67 (21)

When evaluating the adequacy of current guidelines, 11% believed they are comprehensive and useful, 61% felt they are helpful but could be improved, 13% said they lack sufficient evidence, 8% considered them outdated and 6% reported being unfamiliar with them altogether (Table 4). Regarding the existence of a consensus or strong evidence guiding rehabilitation protocols for non‐operative FTSD management, 66% of respondents reported that they believe there is no consensus or strong evidence, 21% were unsure and only 13% believed that a consensus or strong evidence currently exists (Table 4).

### Influence of demographic variables on management strategies and perceptions

Chi‐square tests and regression analyses were conducted to evaluate how respondents' age and geographical location may influence FTSD management strategies and perceptions on evidence and guidelines. Country‐level data were grouped by continent (North America and Europe) due to the small sample size per individual country precluding meaningful and statistically robust comparisons. Since most respondents (95.1%) were from the same speciality (orthopaedic surgeons), subgroup analyses by speciality were not conducted.

### Age

#### Type of stabilization

Age was significantly associated with stabilization choice (*p* = 0.006). Younger respondents, particularly those under 45, showed a stronger preference for arthroscopic soft tissue stabilization, while older respondents, particularly those over 55, showed a preference towards open soft tissue stabilization and open or arthroscopic bony stabilization.

#### Use of specific guidelines or protocols

Age was also significantly associated with the use of clinical guidelines when managing FTSDs (*p* = 0.031). Respondents in the 36–45 age group were more likely to adhere to specific guidelines, while older respondents (>55) reported relying more on personal experience rather than formal guidelines.

### Geographical location

#### Imaging preferences

European respondents were more likely to use MRI compared to North American respondents (*p* = 0.008), whereas North Americans were more likely to use X‐rays (*p* = 0.007).

#### Immobilization practices

Both European and North American respondents most commonly recommend 1–3 weeks of immobilization following an FTSD; however, European respondents were significantly more likely to consistently choose the 1‐ to 3‐week duration compared to the North American respondents (*p* = 0.01). In contrast, North American respondents demonstrated more variability, leaning more towards less than one week.

#### Limiting external rotation

Respondents from North America were significantly more likely to limit external rotation during the initial phase of physiotherapy compared to respondents from Europe (*p* < 0.0001).

#### Perceptions on evidence and guidelines

When asked about the evidence supporting primary arthroscopic stabilization for FTSDs, respondents in North America were significantly more likely than those in Europe to agree or strongly agree that sufficient evidence exists (*p* < 0.001). North Americans were also more likely to believe that there is a consensus or strong evidence guiding rehabilitation protocols for non‐operative FTSD management (*p* < 0.001).

## DISCUSSION

### Summary of key findings

The majority of respondents were fellowship‐trained orthopaedic surgeons between the ages of 35 and 55 years old and were based in either North America or Europe. The findings of this study indicate that the most common treatment approach following an FTSD is a period of immobilization of 1–3 weeks, after which physical therapy is typically initiated. The majority of respondents reported utilizing a combination of strengthening, range of motion, and proprioceptive exercises as part of their rehabilitation protocol. Only about half of the respondents reported using guideline‐based management, with many, particularly those over 55 years old, continuing to base their practices on personal experience and training rather than standardized protocols.

The most frequently used imaging modalities used to evaluate FTSDs were X‐ray and MRI. Interestingly, imaging preferences varied based on geographic location, with North American respondents more likely to use X‐ray and European respondents more likely to use MRI. However, it is important to note that the survey did not ask respondents to specify the clinical scenarios or indications for ordering different imaging, limiting the ability to provide detailed context on when MRI vs X‐ray is favoured. The top three most important factors influencing surgical referral for an FTSD were the presence of bony injury or anterior GBL, patient age and participation in contact or collision sports. The most common surgical procedure selected to treat FTSD was arthroscopic soft tissue repair. Notably, younger respondents were more likely to select this option, with older respondents leaning more towards open or bony repairs. The presence of an engaging Hill‐Sachs lesion was the most common indication for the addition of remplissage to a soft tissue repair.

Regarding perceptions on evidence and guidelines, most respondents expressed confidence in the evidence supporting primary arthroscopic stabilization for FTSDs. To stay current with the latest evidence and guidelines on shoulder dislocation treatment, respondents primarily relied on peer‐reviewed journals and professional conferences. Most respondents found the guidelines to be adequate but noted that there is room for improvement. There was also a perceived lack of consensus or strong evidence regarding rehabilitation protocols for non‐operative FTSD management.

### Relation to other work

Our findings align with existing literature that highlights both variability and emerging trends towards consensus in the management of FTSDs [13]. Historically, the management of FTSDs has been a subject of debate, with no clear consensus on optimal treatment strategies [1]. The primary divide has been between non‐operative management (immobilization and rehabilitation) and operative management [5, 13].

While the debate between operative and non‐operative approaches remains ongoing, there is also variability within each management option. For non‐operative treatment, factors such as immobilization duration, timing of physical therapy initiation and the choice of rehabilitation exercises vary among practitioners [12]. Similarly, among those favouring operative management, differences exist regarding the choice between arthroscopic or open stabilization, soft tissue or bony repair and the inclusion of additional procedures like remplissage, as previously reported in the literature [3]. The results of this survey demonstrated this existing variability in practice; however, it is important to note the emerging trend towards arthroscopic soft tissue stabilization as the optimal operative treatment choice for this patient population.

Ultimately, the treatment choice should consider individual circumstances, given the associated risks and benefits of both approaches [9]. This individualized approach is consistent with studies that emphasize the importance of tailored treatment plans, particularly when there is no clear consensus on best practices [9]. Based on the results of this study, the presence of bony injury or anterior GBL, patient age, and participation in contact or collision sports are among the most important factors influencing surgical referral for an FTSD.

### Implications for clinical and research

This study highlights the need for the development of robust, evidence‐based guidelines to standardize the management of FTSDs. Despite some variability, the general trend towards consensus on key practices demonstrates an opportunity to further consolidate and disseminate best practices. Developing such guidelines would facilitate consistency in treatment approaches and improve patient outcomes by minimizing discrepancies between healthcare settings. The differences observed based on age and geographical location emphasize the need to develop guidelines with standardized approaches based on the best available evidence.

Variability within both surgical and non‐surgical approaches suggests that future research should address not only the choice between operative and non‐operative management options but also the nuances within each approach. Other important considerations necessitating further research include incorporating patient preferences and risk stratification tools in the decision‐making process in order to provide personalized treatments tailored to individual patient profiles.

### Strengths and limitations

The survey underwent a rigorous review process to ensure both face and content validity, enhancing the reliability of the collected data. Additionally, robust statistical analyses were employed to evaluate trends based on age and geographical location, allowing for a comprehensive understanding of the observed variations. The global distribution of the survey further increased the generalizability of the findings, making them more applicable to diverse populations.

The study also had several limitations. One notable limitation was the relatively small sample size, which may affect the statistical power of the analyses. Additionally, because multiple statistical comparisons were performed without correction, the possibility of type I error must be considered when interpreting the results. Although the survey was designed to have a global reach, it was only available in English, limiting participation to English speakers and potentially introducing language bias. In addition, while the total number of members from each organization that confirmed distribution of the survey was obtained (Appendix SB), accurately calculating response rates remained difficult, as some organizations that did not confirm distribution may have still circulated the survey. Furthermore, since the survey did not collect respondents' specific organizational affiliation, response rate estimates would require assuming that respondents' countries of practice align with specific organizations. Also, the survey did not capture specific respondent characteristics, such as whether they were academic‐affiliated, community‐based or rural. Finally, the use of an electronic survey format may have introduced response bias, as it inherently limits participation to individuals with regular access to computers.

## CONCLUSION

While variability in the management of FTSDs persists, the results of this study demonstrate emerging trends towards consensus on critical factors for consideration in the management of such patients. However, despite the prevalence of guideline‐based management, a substantial proportion of respondents, particularly those over 55 years old, continue to base their practices on personal experience and training rather than standardized protocols. The development and dissemination of evidence‐based guidelines remain essential to standardize clinical practices and optimize patient outcomes.

## AUTHOR CONTRIBUTIONS

Each author listed on this work fulfils the ICMJE criteria for authorship.

## CONFLICT OF INTEREST STATEMENT

Author Xinning (Tiger) Li has received consulting and advisory fees from FH ORTHO. Author Peter MacDonald has received consulting and advisory fees from Arthrex Inc. and provides educational support for Conmed, Arthrex, Stryker and Smith and Nephew. The remaining authors declare no conflicts of interest.

## ETHICS STATEMENT

All procedures in this study were approved by the Hamilton Integrated Research Ethics Board (Project ID: 17955).

## Supporting information

Appendix SA. FTSD Sport Doc Survey_Questionnaire.

Appendix SB. List of sport med organizations.

## Data Availability

The data that support the findings of this study are available from the corresponding author upon reasonable request.
