# Supplementary material for: Management of first‐time shoulder dislocations: A survey of sport medicine physician perceptions
Source: Knee Surg Sports Traumatol Arthrosc. 2026 Feb 6;34(4):1421–30. doi: 10.1002/ksa.70297 (PMC13037362; doi:10.1002/ksa.70297)
Supplement: Supplementary file 1 — Appendix SA. FTSD Sport Doc Survey_Questionnaire. [file KSA-34-1421-s001.docx]

**Appendix A.** Complete Study Survey

**Title of study:** Management of First-Time Shoulder Dislocations: A Survey of Sport Physician Perceptions

1. How old are you?
   1. 18-24 years old
   2. 25-35 years old
   3. 36-45 years old
   4. 46-55 years old
   5. >55 years old
2. How many years have you been in independent practice?
   1. <5 years
   2. 5-10 years
   3. 11-20 years
   4. 21-25 years
   5. >25 years
3. What is your primary specialty?
   1. Primary Care Sports Medicine
   2. Orthopedic Surgery
   3. Physical Medicine and Rehabilitation
   4. Emergency Medicine
   5. Physician assistant/nurse practitioner
   6. Other (please specify)
4. Have you completed any formal post-graduate subspecialty fellowship training in sport medicine or shoulder/elbow surgery?
   1. Yes
      1. Sports Medicine Fellowship
      2. Shoulder and Elbow Fellowship
   2. No
5. Where is your practice located?

[drop-down list of all countries in alphabetical order]

1. What population(s) do you primarily work with? (Select all that apply)
   1. Paediatrics
   2. General outpatient orthopaedic patients
   3. Recreational athletes
   4. High school athletes
   5. Collegiate athletes
   6. Professional/elite athletes
   7. Seniors
   8. Other (specify): ____
2. Do you treat patients who have experienced shoulder dislocations?
   1. Yes
   2. No

*If you answered “no” to question #7, you will be skipped ahead to question #18.*

**Note***:* The population of interest includes patients under 30 years old. Please only consider this population when answering the following questions.

1. How many **first-time shoulder dislocation (FTSD)** cases do you manage annually, whether surgically or conservatively?
   1. 0
   2. 1-5
   3. 6-10
   4. 11-20
   5. 20+
2. How soon do you typically see first time dislocators following their dislocation?
   1. Less than one week after the dislocation
   2. 1-2 weeks after the dislocation
   3. 2-6 weeks after the dislocation
   4. 6-12 weeks after the dislocation
   5. More than 12 weeks after the dislocation
3. What duration of immobilization do you typically recommend after a FTSD?
   1. No immobilization
   2. Less than 1 week
   3. 1-3 weeks
   4. 3-6 weeks
   5. More than 6 weeks
4. When do you typically initiate physical therapy following a FTSD?
   1. Immediately after reduction
   2. After a period of immobilization
   3. Only if there are signs of instability after immobilization
   4. I do not typically refer to physical therapy
   5. Other (please specify)
5. What type of physical therapy protocol do you most commonly recommend for FTSDs?
   1. Strengthening exercises
   2. Range of motion exercises
   3. Proprioceptive training
   4. A combination of all of the above
   5. I do not recommend physical therapy
6. Do you limit external rotation (ER) in the initial phase of your physiotherapy protocols for FTSDs?
   1. Yes
   2. No
7. Do you use any specific guidelines or protocols to guide your treatment of FTSDs?
   1. Yes, I follow specific guidelines (e.g., American Academy of Orthopaedic Surgeons, European Society of Sports Traumatology, Knee Surgery and Arthroscopy)
   2. Yes, but I use a combination of different guidelines
   3. No, I base my treatment on personal experience and training
   4. No, I rely on the latest research literature
   5. Other (please specify)
8. Which imaging modalities do you use to evaluate a FTSD in a majority of patients? (Select all that apply)
   1. X-ray
   2. MRI
   3. MR Arthrogram
   4. CT scan
   5. Ultrasound
   6. I do not use imaging routinely
9. Please select the **top 3** factors from the options below that, in your opinion, are the most important when deciding whether to refer a patient to surgery/surgical evaluation following a FTSD.
   1. Age of the patient
   2. Involvement in contact/collision sports
   3. Presence of bony injury or anterior glenoid bone loss
   4. Degree/persistence of instability
   5. Failure of conservative management
   6. Patient preference
10. Do you perform surgery on FTSDs?
    1. Yes
    2. No

17 A) In surgical cases for FTSDs with minimal to no bone loss present, which type of stabilization do you perform?

- 1. Open soft tissue stabilization
  2. Arthroscopic soft tissue stabilization
  3. Open or arthroscopic bony stabilization

17 B) In surgical cases of soft tissue stabilization for FTSDs, when do you decide to add in the Remplissage procedure to the Bankart repair? (Select all that apply)

- 1. Engaging Hill Sachs lesion
  2. Any Hill Sachs lesion
  3. Glenoid bone loss <10%
  4. Glenoid bone loss >10%
  5. Apprehension/reduced range of motion
  6. I add Remplissage to every case
  7. I never do Remplissage

1. Please rate the extent to which you agree with the following statement: “In my opinion, there is sufficient evidence supporting primary arthroscopic stabilization for the treatment of FTSDs.”
   1. Strongly agree
   2. Agree
   3. Neither agree nor disagree
   4. Disagree
   5. Strongly disagree
2. How do you stay current with the latest evidence and guidelines regarding shoulder dislocation treatment? (Select all that apply)
   1. Continuing medical education (CME) courses
   2. Professional conferences
   3. Peer-reviewed journals
   4. Online medical resources and databases
   5. Professional networks and consultations
   6. Other (please specify)
3. Do you believe that current guidelines adequately address the management of FTSDs?
   1. Yes, they are comprehensive and useful
   2. Yes, but there is room for improvement
   3. No, they are outdated
   4. No, they lack sufficient evidence
   5. I am not familiar with current guidelines
4. Do you believe there is a consensus or strong evidence regarding rehabilitation protocols to guide non-operative management of FTSDs?
   1. Yes
   2. No
   3. Unsure
