# Supplementary material for: Management of first‐time shoulder dislocations: A survey of sport medicine physician perceptions
Source: Knee Surg Sports Traumatol Arthrosc. 2026 Feb 6;34(4):1421–30. doi: 10.1002/ksa.70297 (PMC13037362; doi:10.1002/ksa.70297)
Supplement: Supplementary file 2 — Appendix SB. List of sport med organizations. [file KSA-34-1421-s002.pdf]

Organization	Number of Members	Survey Distribution Status
American Orthopedic Association for Sports Medicine (AOSSM)	3944	Distributed
American Shoulder and Elbow Surgeons (ASES)	1411	Distributed
Arthroscopy Association of Canada (AAC)	157	Distributed
Arthroscopy Association of North America (AANA)	5000	Distributed
Brazilian Society of Shoulder and Elbow Surgery (SBCOC)	1200	Distributed
Dutch Shoulder and Elbow Society (DSES)	200	Distributed
French Society of Shoulder and Elbow Surgery (SFA)	500-1000	Distributed
Hungarian Orthopaedic Association (MOT)	60	Distributed
Italian Society of Orthopaedics Traumatology (SIOT)	3357	Distributed
Italian Society of Shoulder and Elbow Surgery (SICSeG)	200	Distributed
Polish Shoulder and Elbow Society (PTBL)	110	Distributed
Portuguese Shoulder and Elbow Society (SPOC)	140	Distributed
Saudi Society for Shoulder and Elbow Surgery	80	Distributed
German Society for Orthopaedics and Trauma (DGOU)		Deferred to Partner Organization
South African Orthopaedic Association (SAOA)		Deferred to Partner Organization
American Medical Society for Sports Medicine (AMSSM)		Declined: Research team must have a member of the organization.
Australian Orthopaedic Association (AOA)		Declined: Research team must have a member of the organization.
European Society for Surgery of the Shoulder and Elbow (ESSSE/SECEC)		Declined: Research team must have a member of the organization.
International Society of Arthroscopy, Knee Surgery and Orthopaedic Sports Medicine (ISAKOS)		Declined: Research team must have a member of the organization.
Japanese Orthopaedic Association (JOA)		Declined: Distribution prohibited according to organizational policies.
Sports Medicine Australia (SMA)		Declined: Research team must have a member of the organization.
Africa Union of Sports Medicine (UAMS)		No Response
Albanian Society for Trauma and Emergency Surgery (ASTES)		No Response
American Academy of Orthopaedic Surgeons (AAOS)		No Response
American College of Sports Medicine (ACSM)		No Response
Argentine Association of Shoulder and Elbow Surgery (AAHC)		No Response
Asia-Pacific Knee, Arthroscopy and Sports Medicine Society (APKASS)		No Response
Asia-Pacific Shoulder and Elbow Society (APSES)		No Response
Asian Federation of Sports Medicine (AFSM)		No Response
Asian Pacific Hand & Upper Limb Society (APH&ULS)		No Response
Austrian Society for Orthopaedics and Orthopaedic Surgery (ÖGO)		No Response
Beijing Institute of Sports Medicine (BISM)		No Response
Belarus Republic Scientific Medical Society of Trauma and Orthopaedic Surgeons (BRSMTOS)		No Response
Belgian Royal Society of Orthopaedic Surgery and Traumatology (SORBCOT)		No Response
Belgian Society of Orthopaedics and Traumatology (BVOT)		No Response
Brazilian Orthopaedic Association (SBOT)		No Response
Brazilian Society of Exercise and Sports Medicine (SBMEE)		No Response
Brazilian Sports Medicine Society (SBRATE)		No Response
British Association of Sport and Exercise Medicine (BASEM)		No Response
British Association of Sport and Exercise Sciences (BASES)		No Response
British Orthopaedic Association (BOA)		No Response
Canadian Academy of Sport and Exercise Medicine (CASEM)		No Response
Chiliean Shoulder and Elbow Society		No Response
Chiliean Society of Orthopedics and Traumatology-Shoulder and Elbow Committee (SChOT)		No Response
Chinese Shoulder & Elbow Society (CSES)		No Response
Colombian Association of Sports Medicine (AMEDCO)		No Response
Czech Society for Orthopaedics and Traumatology (CSOT)		No Response
Danish Association of Sports Medicine (DIMS)		No Response
Danish Orthopaedic society (DOS)		No Response
Danish Soceity for Shoulder and Elbow Surgery (DSSAK)		No Response
Dutch Association of Sports Medicine (VSG)		No Response
Ecuadorian Shoulder and Elbow Society (ESECUHC)		No Response
Egyptian Shoulder Surgery Association (EGSSA)		No Response
Estonia Orthopaedic Society (EOS)		No Response
European College of Sports Science (ECSS)		No Response
European Society for Shoulder and Elbow Rehabilitation (EUSSER)		No Response
European Society of Sports Traumatology, Knee Surgery & Arthroscopy (ESSKA)		No Response
Finnish Orthopaedic Association (FOA)		No Response
French Society of Orthopaedic Surgery and Traumatology (SOFCOT)		No Response
German Society for Shoulder and Elbow Surgery (DVSE)		No Response
German Society for Sports Medicine and Prevention (DGSP)		No Response
Hellenic Association of Orthopaedic Surgery and Traumatology (EEXOT)		No Response
Hong Kong Association of Sports Medicine and Sports Science (HKASMSS)		No Response
Hungarian Society of Shoulder and Elbow Surgery (MVKE)		No Response
Indian Association of Sports Medicine (IASM)		No Response
Indian Orthopaedic Association (IOA)		No Response
International Federation of Sports Medicine (FIMS)		No Response
International Society of Orthopaedic Surgery and Traumatology (SICOT)		No Response
Irish Orthopaedic Association (IOA)		No Response
Irish Shoulder and Elbow Society (ISES)		No Response
Italian Federation of Sports Medicine (FMSI)		No Response
Japan Sports Orthopedic Association (JSOA)		No Response
Japanese Shoulder Society (JSS)		No Response
Korean Shoulder and Elbow Society (KSES)		No Response
Lebanese Orthopaedic Society (LOS)		No Response
Lithuanian Society of Orthopaedics and Traumatology (LSOT)		No Response
Mexican Society of Shoulder and Elbow Surgeons (SMCHC)		No Response
New Zealand Orthopaedic Association (NZOA)		No Response
New Zealand Sports Medicine (NZSM)		No Response
Norwegian Orthopaedic Association (NOF)		No Response
Norwegian Society for Surgery of the Shoulder and Elbow (NFSA)		No Response
Orthopaedic and Traumatology Surgeons Association of Bosnia and Herzegovina (OSTABH)		No Response
Pan Arab Orthopedic Association (PAOA)		No Response
Pan-Arabic Shoulder and Elbow Society (PASES)		No Response
Peruvian society of orthopedics and traumatology (SPOT)		No Response
Polish Orthopaedic and Traumatologic Society (PTOiTr)		No Response
Romanian Society of Orthopaedics and Traumatology (SOROT)		No Response
Saudi Orthopaedic Association (SOA)		No Response
Shoulder and Elbow Society of India (SESI)		No Response
South African Society of Endoscopic Surgeons (SASES)		No Response
South African Sports Medicine Association (SASMA)		No Response
Spanish Society of Shoulder and Elbow Surgery (SECHC)		No Response
Spanish Society of Sports Medicine		No Response
Sports and Exercise Physiotherapy New Zealand (SEPNZ)		No Response
Swedish Shoulder Surgery Society (SSAS)		No Response
Swiss Trauma Society (STS)		No Response
Taiwan Shoulder and Elbow Society (TSES)		No Response
The Finnish Society for Shoulder and Elbow Surgery (SOKY)		No Response
Turkish Society of Orthopaedics and Traumatology (TOTBID)		No Response
